# Expression patterns of novel immunotherapy targets in intermediate- and high-grade lung neuroendocrine neoplasms

**DOI:** 10.1007/s00262-024-03704-7

**Published:** 2024-05-02

**Authors:** Bence Ferencz, Klára Török, Orsolya Pipek, János Fillinger, Kristóf Csende, András Lantos, Radoslava Černeková, Marcel Mitták, Jozef Škarda, Patricie Delongová, Evelyn Megyesfalvi, Karin Schelch, Christian Lang, Anna Solta, Kristiina Boettiger, Luka Brcic, Jörg Lindenmann, Ferenc Rényi-Vámos, Clemens Aigner, Judit Berta, Zsolt Megyesfalvi, Balázs Döme

**Affiliations:** 1 Department of Thoracic Surgery, Semmelweis University and National Institute of Oncology, Budapest, Hungary; 2 National Koranyi Institute of Pulmonology, Budapest, Hungary; 3 Department of Physics of Complex Systems, Eotvos Lorand University, Budapest, Hungary; 4 Department of Pulmonary Diseases and Tuberculosis, University Hospital Ostrava and Faculty of Medicine, University of Ostrava, Ostrava, Czech Republic; 5 Surgical Clinic, University Hospital Ostrava and Faculty of Medicine, University of Ostrava, Ostrava, Czech Republic; 6 Medical Faculty, Institute of Clinical and Molecular Pathology, Palacky University Olomouc, Olomouc, Czech Republic; 7 Department of Pathology, University Hospital Ostrava and Faculty of Medicine, University of Ostrava, Ostrava, Czech Republic; 8 Department of Clinical Pharmacology, National Institute of Oncology, Chest and Abdominal Tumors Chemotherapy “B”, Budapest, Hungary; 9 Department of Thoracic Surgery, Comprehensive Cancer Center Vienna, Medical University of Vienna, Waehringer Guertel 18-20, 1090 Vienna, Austria; 10 Center for Cancer Research, Medical University of Vienna, Vienna, Austria; 11 Division of Pulmonology, Department of Medicine II, Medical University of Vienna, Vienna, Austria; 12 Diagnostic and Research Institute of Pathology, Medical University of Graz, Graz, Austria; 13 Division of Thoracic and Hyperbaric Surgery, Department of Surgery, Medical University of Graz, Graz, Austria; 14 National Institute of Oncology and National Tumor Biology Laboratory, Budapest, Hungary; 15 Department of Translational Medicine, Lund University, Lund, Sweden

**Keywords:** Lung neuroendocrine neoplasm, Immunotherapy target, Immune phenotype, Immunohistochemistry

## Abstract

**Background:**

Advancements in immunotherapeutic approaches only had a modest impact on the therapy of lung neuroendocrine neoplasms (LNENs). Our multicenter study aimed to investigate the expression patterns of novel immunotherapy targets in intermediate- and high-grade LNENs.

**Methods:**

The expressions of V-domain Ig suppressor of T cell activation (VISTA), OX40L, Glucocorticoid-induced TNF receptor (GITR), and T cell immunoglobulin and mucin domain 3 (TIM3) proteins were measured by immunohistochemistry in surgically resected tumor samples of 26 atypical carcinoid (AC), 49 large cell neuroendocrine lung cancer (LCNEC), and 66 small cell lung cancer (SCLC) patients. Tumor and immune cells were separately scored.

**Results:**

Tumor cell TIM3 expression was the highest in ACs (*p* < 0.001), whereas elevated tumor cell GITR levels were characteristic for both ACs and SCLCs (*p* < 0.001 and *p* = 0.011, respectively). OX40L expression of tumor cells was considerably lower in ACs (vs. SCLCs; *p* < 0.001). Tumor cell VISTA expression was consistently low in LNENs, with no significant differences across histological subtypes. ACs were the least immunogenic tumors concerning immune cell abundance (*p* < 0.001). Immune cell VISTA and GITR expressions were also significantly lower in these intermediate-grade malignancies than in SCLCs or in LCNECs. Immune cell TIM3 and GITR expressions were associated with borderline prognostic significance in our multivariate model (*p* = 0.057 and *p* = 0.071, respectively).

**Conclusions:**

LNEN subtypes have characteristic and widely divergent VISTA, OX40L, GITR, and TIM3 protein expressions. By shedding light on the different expression patterns of these immunotherapy targets, the current multicenter study provides support for the future implementation of novel immunotherapeutic approaches.

**Supplementary Information:**

The online version contains supplementary material available at 10.1007/s00262-024-03704-7.

## Introduction

Lung neuroendocrine neoplasms (LNENs) account for one fifth of all pulmonary malignancies and comprise four histological subtypes with different clinical and biological characteristics [1–5]. Pulmonary carcinoids represent 10% of LNENs and comprise two major subtypes: typical and atypical carcinoids. Typical carcinoids are highly differentiated tumors that are predominantly curable by surgical resection. Accordingly, the five-year overall survival (OS) rate of these patients exceeds 80% and the recurrence rate is low [6]. Meanwhile, atypical carcinoids (ACs) are moderately differentiated, intermediate-grade tumors with greater metastatic potential compared to typical carcinoids, and with a five-year OS rate of 50%. As a result of their aggressive nature, ACs often necessitate adjuvant chemotherapy after surgical resection [7–9]. Large cell neuroendocrine lung cancer (LCNEC) is a poorly differentiated, high-grade tumor with a complex biology that shares similarities with both small cell lung cancer (SCLC) and non-SCLC (NSCLC). Therapeutic approaches often overlap with management protocols for SCLC, the most lethal lung carcinoma [10]. SCLC is a heterogeneous malignancy characterized by genomic instability, early metastasis, and a rapid proliferation rate. As this tumor type is often diagnosed at an advanced stage, curative-intent surgery is rarely performed, and treatment usually consists of chemo-immunotherapy with or without radiation [11, 12].

Targeted therapy and immunotherapy have revolutionized the management protocols of NSCLC patients, yet the therapeutic advancements in LNENs are poor [13–17]. This is primarily due to the lack of targetable driver mutations and to the conflicting results of immunotherapy-related trials [12]. The tumor immune microenvironment (TIM) has been shown to play a key role in the efficacy of immune checkpoint inhibitors (ICIs) [18, 19]. In this context, assessment of TIM, characteristic immune checkpoints, and specific immune patterns of LNENs is an essential step in understanding and improving the efficacy of currently used and forthcoming immunotherapeutic approaches.

In addition to the well-studied immune checkpoint molecules such as programmed cell death protein 1 (PD-1), programmed cell death-ligand 1 (PD-L1), and cytotoxic T lymphocyte-associated protein 4 (CTLA-4), other molecules with relevant functions in antitumor immunity are worth investigating [20–23]. One of these molecules of potential clinical importance is the V-domain Ig suppressor of T cell activation (VISTA), a transmembrane protein that inhibits the effector function of T cells. VISTA is usually highly expressed in tumor-infiltrating lymphocytes, leading to a decreased antitumoral immune response [24]. High VISTA expression has been described in various malignancies such as melanoma, NSCLC, and pleural mesothelioma [25–27]. OX40L (CD252) is the ligand of the OX40 (CD134) receptor and is usually expressed by antigen-presenting cells (APCs) such as dendritic cells or macrophages [28]. Importantly, some studies have highlighted that agonists of OX40 and OX40L can enhance antitumoral immunity [29]. Glucocorticoid-induced TNF receptor (GITR) is also a transmembrane protein and plays a pivotal role in the regulation of effector T cells. Importantly, its activation can regulate antitumoral immune response [30, 31]. T cell immunoglobulin and mucin domain 3 (TIM3) is an immunoregulatory protein of T lymphocytes, myeloid cells, and several tumor cells (TCs) (e.g., melanoma, breast, and kidney cancer). Since TIM3 promotes the development of several tumors by suppressing antitumoral immunity, blockage of the TIM3 pathway might be a promising therapeutic approach [32, 33]. Although the druggability of these novel immunotherapy targets has already been validated in preclinical settings, their therapeutic relevance has not yet been assessed in LNEN patients [28, 34–37]. Nevertheless, elucidating their expression pattern in human LNENs should be among the first steps in planning specific clinical trials evaluating the efficacy of particular immunotherapeutics directed against VISTA, OX40L, GITR, and TIM3.

In order to provide insights into the applicability of immunotherapy in highly malignant LNENs, the current study aimed to investigate the expression levels and distribution patterns of immunologic markers of potential therapeutic relevance in SCLC, AC, and LCNEC patients. Notably, to provide a comprehensive overview of immunologic marker expression within the whole tumor, the study was conducted using surgically resected specimens.

## Methods

### Study population and treatment

A total of 141 surgically treated LNEN patients from the following four Central European centers were included: National Koranyi Institute of Pulmonology (Budapest, Hungary), National Institute of Oncology (Budapest, Hungary), Medical University of Graz (Graz, Austria), and Palacky University Olomouc (Olomouc, Czech Republic). Of these patients, 66, 49, and 26 were diagnosed with SCLC, LCNEC, and AC, respectively. Concerning the enrollment period, SCLC samples originated from between 1997 and 2020, whereas LCNEC and AC FFPE blocks were all created in 2016–2021 and 2008–2019, respectively. Only whole-tissue specimens were included to avoid bias due to intratumoral heterogeneity. To achieve a representative cohort size, all individuals who underwent surgical resection for LNEN in the participating institutions were included. Further inclusion criteria consisted of adequate tumor content (> 20% of all cells) in FFPE blocks as defined by an expert pathologist. Clinicopathological data were collected retrospectively from the medical records of each center. The study was conducted in accordance with the guidelines of the Helsinki Declaration of the World Medical Association and with the approval of the national-level Ethics Committee of each participating country. Patient identifiers were removed after clinical data collection to ensure patient pseudonymity. The requirement for written informed consent was waived due to the study’s retrospective nature. All patients underwent lung resection surgery, and adjuvant chemo- and/or radiotherapy was administered when necessary. Each therapeutic approach was applied in accordance with the contemporary National Comprehensive Cancer Network (NCCN) guidelines.

### Patient samples and immunohistochemistry

Prior to enrolment, all slides were re-evaluated by a board-certified pathologist to confirm the diagnosis of LNEN. Next, tissue samples were analyzed for the expression of the four TIM markers including TIM3, VISTA, GITR, and OX40L. Due to the low tissue quantity of samples, in 21 SCLC cases, only VISTA expression was measured. The specific antibodies against these markers are summarized in Supplementary Table S1. To assess the quality and reliability of the older (> 15 years) FFPE blocks concerning SCLC patients, these samples were also stained with routinely used diagnostic antibodies against CD56 [38] and Ki-67 [39]. The degree of immune infiltration was assessed by analyzing CD3 expression. Immunohistochemistry (IHC) staining was performed according to the recommended staining protocols using Ventana BenchMark Ultra IHC/ISH System. (Roche diagnostics, Basel, Switzerland). In brief, after deparaffinization and incubation with the primary antibody, the secondary antibody was applied for one hour at room temperature. Visualization of the expression levels was achieved with Liquid DAB and Substrate Chromogen System, and sections were counterstained with hematoxylin. Of note, the staining protocol was validated by human tonsils as positive tissue controls. Expression of the given marker was examined blinded to clinical data by two experienced independent lung pathologists. All slides were digitally scanned using PANNORAMIC 250 Flash III (3DHISTECH Ltd., Budapest, Hungary); sections were examined and evaluated by using CaseViewer 2.4 (3DHISTECH Ltd., Budapest, Hungary). Providing an overview of generalized marker expression across the tumor area is essential for biomarker discovery[40]. Therefore, during pathological evaluation, we determined the percentage of positive TCs in at least 20 randomly selected areas at 20 × and 40 × magnification. Two experienced pulmonary pathologists performed the evaluation process, and if a discrepancy of > 20% occurred in their results, a third pulmonary pathologist was also involved.

TCs were evaluated separately from immune cells (ICs). In the case of TCs, the ratio of positive cells to all TCs was also quantified. Similarly, the ratio of ICs showing positive staining and the ratio of total immune infiltrates in a given sample were determined. It should be emphasized that manual analysis of each marker was preferred in this study since software-based evaluation still bears many limitations, even for antibodies used in routine diagnostics.

### Statistical analysis

All statistical analyses were performed in R version 4.2.1 (R Foundation for Statistical Computing, Vienna, Austria). Associations between histological subtypes and clinicopathological characteristics were assessed by Fisher’s exact tests and Kruskal–Wallis rank sum tests for categorical and continuous variables, respectively. Adjustment for multiple comparisons was achieved with the Bonferroni-method. Marker expression levels and clinicopathological parameters were compared with Wilcoxon signed-rank tests with Bonferroni correction. Hierarchical clustering of samples based on the expression levels was performed with the ComplexHeatmap R package (version 2.10.0). The distance matrix was calculated using Manhattan distance measure and the dendrograms were created using the ward.D clustering method. Pearson-correlation coefficients (R) were calculated between expression levels, with p values corrected for multiple comparisons with the Bonferroni-method. To investigate which expression levels are most indicative of LNEN subtype, a principal component analysis (PCA) was performed (with the factoextra R package (version 1.0.7)) to find linear combinations (“principal components” (PCs)) of the measured variables (expression levels) that most effectively explain the variance in the data. Clinical factors having a prognostic relevance for OS were determined by univariate Kaplan–Meier analysis. Survival curves of different patient subgroups were compared with log-rank tests. P values were not adjusted for multiple testing. To assess the prognostic relevance of different marker expression, patients were divided into “low” (i.e., median or below-median) vs. “high” (i.e., above-median) expressing groups based on the median expression value of each marker. Guided by the results of univariate analyses, a multivariate Cox-regression model was fitted to the data.

## Results

### Patient and sample characteristics

Clinicopathological features of included patients according to LNEN histological subtypes are summarized in Table 1. Most SCLC and LCNEC patients were smokers, whereas the majority of individuals diagnosed with AC were never-smokers (*p* < 0.001). SCLC tumors tended to be centrally located, contrasting the peripheral localization of LCNEC (*p* < 0.001). Univariate models were applied to evaluate survival outcomes considering clinicopathological characteristics. Diabetes (*p* = 0.019), histological subtype (*p* = 0.035), vascular involvement (*p* = 0.0063), as well as T and N stages (*p* = 0.078 and 0.044, respectively) were all relevant factors for survival (Supplementary Fig. 1). As for the antibodies used for quality check of the older (> 15 years) FFPE samples, we found strong positivity with CD56 and moderate positivity (associated with reduction of immunosignal intensity) with Ki-67 (Supplementary Fig. 2). Notably, expression patterns of TIM3, VISTA, GITR, and OX40L did not differ statistically significantly between the older (> 15 years) and newer (≤ 15 years) blocks.

**Table 1:** Clinicopathological characteristics of the study population

		Total	AC	LCNEC	SCLC	*p* value
Total number of patients		141	26	49	66	
Gender	N/A	9	0	6	3	0.771^1^
	Male	66 (50%)	11 (43.2%)	23 (53.3%)	32 (50.8%)	
	Female	66 (50%)	15 (57.7%)	20 (46.5%)	31 (49.2%)	
Age	N/A	10	0	7	3	0.333^2^
	Median (Range)	65 [33–79]	62.5 [33–79]	64.5 [41–78]	65 [44–78]	
Smoking status	N/A	36	2	13	21	** < 0.001**^**1**^
	Never	21 (20.0%)	14 (58.3%)	3 (8.3%)	4 (8.9%)	
	Ex	44 (41.9%)	6 (25.0%)	15 (41.7%)	23 (51.1%)	
	Current	40 (38.1%)	4 (16.7%)	18 (50.0%)	18 (40.0%)	
COPD	N/A	13	0	8	5	**0.049**^**1**^
	No	77 (60.2%)	22 (84.6%)	21 (51.2%)	34 (55.7%)	
	Yes	51 (39.8%)	4 (15.4%)	20 (48.8%)	27 (44.3%)	
Hypertension	N/A	12	0	7	5	0.495^1^
	No	55 (42.6%)	10 (38.5%)	15 (35.7%)	30 (49.2%)	
	Yes	74 (57.4%)	16 (61.5%)	27 (64.3%)	31 (50.8%)	
Diabetes	N/A	12	0	7	5	0.495^1^
	No	104 (80.6%)	22 (84.6%)	36 (85.7%)	46 (75.4%)	
	Yes	25 (19.4%)	4 (15.4%)	6 (14.3%)	15 (24.6%)	
Tumor localization (central/peripheral)	N/A	22	0	10	12	** < 0.001**^**1**^
	Central	51 (42.9%)	13 (50.0%)	5 (12.8%)	33 (61.1%)	
	Peripheral	68 (57.1%)	13 (50.0%)	34 (87.2%)	21 (38.9%)	
Tumor localization (upper/lower lobe)	N/A	41	0	7	34	0.333^1^
	Upper lobe	72 (72.0%)	15 (57.7%)	32 (76.2%)	25 (78.1%)	
	Lower lobe	28 (28.0%)	11 (42.3%)	10 (23.8%)	7 (21.9%)	
Necrosis	N/A	22	10	7	5	0.333^1^
	No	43 (36.1%)	6 (37.5%)	11 (26.2%)	26 (42.6%)	
	Yes	76 (63.9%)	10 (62.5%)	31 (73.8%)	35 (57.4%)	
Vascular involvement	N/A	16	1	7	8	0.771^1^
	No	77 (61.6%)	15 (60.0%)	28 (66.7%)	34 (58.6%)	
	Yes	48 (38.4%)	10 (40.0%)	14 (33.3%)	24 (41.4%)	
Peritumoral inflammation	N/A	84	18	15	51	0.310^1^
	0	43 (75.4%)	5 (62.5%)	24 (70.6%)	14 (93.3%)	
	1	9 (15.8%)	3 (37.5%)	5 (14.7%)	1 (6.7%)	
	2	5 (8.8%)	0 (0.0%)	5 (14.7%)	0 (0.0%)	
T	N/A	16	0	9	7	0.227^1^
	1	82 (65.6%)	13 (50.0%)	23 (57.5%)	46 (78.0%)	
	2	25 (20.0%)	7 (26.9%)	11 (27.5%)	7 (11.9%)	
	3	8 (6.4%)	2 (7.7%)	4 (10.0%)	2 (3.4%)	
	4	10 (8.0%)	4 (15.4%)	2 (5.0%)	4 (6.8%)	
N	N/A	43	0	8	35	0.227^1^
	0	53 (54.1%)	12 (46.2%)	28 (68.3%)	13 (41.9%)	
	1	19 (19.4%)	7 (26.9%)	7 (17.1%)	5 (16.1%)	
	2	19 (19.4%)	6 (23.1%)	3 (7.3%)	10 (32.3%)	
	x	7 (7.1%)	1 (3.8%)	3 (7.3%)	3 (9.7%)	
M	N/A	84	10	27	47	0.900^1^
	0	2 (3.5%)	0 (0.0%)	1 (4.5%)	1 (5.3%)	
	1	1 (1.8%)	0 (0.0%)	0 (0.0%)	1 (5.3%)	
	x	54 (94.7%)	16 (100.0%)	21 (95.5%)	17 (89.5%)	

Statistically significant *p* values are marked with bold

*COPD*, Chronic obstructive pulmonary disease; *N/A*, Not available; *AC*, Atypical carcinoid; *LCNEC*, Large cell neuroendocrine lung cancer; *SCLC*, Small cell lung cancer

^1^Fisher’s exact test for count data (adjusted for multiple comparisons)

^2^Kruskal–Wallis rank sum test (adjusted for multiple comparisons)

### Distribution pattern of immunologic markers by TCs

**Fig. 1:**
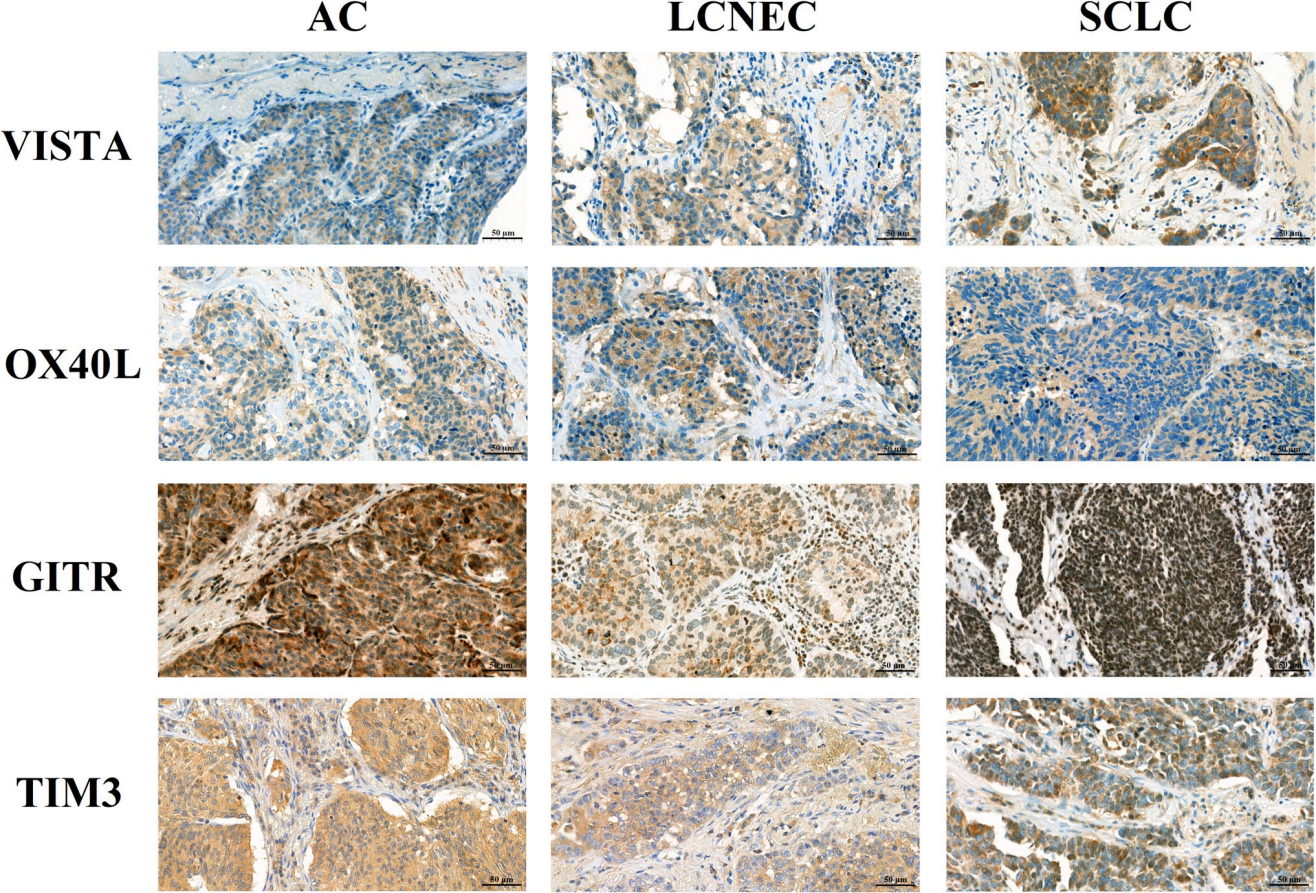
Immunohistochemistry staining of formalin-fixed, paraffin-embedded AC, LCNEC, and SCLC samples with the four immune-related markers. Representative images for TCs with positive staining were captured with a 40 × objective lens. Positive cells were visualized with 3–3’-diaminobenzidine (DAB), and nuclei were labeled with hematoxylin. Scale bar: 50 μm. AC, atypical carcinoid; LCNEC, large cell neuroendocrine lung cancer; SCLC, small cell lung cancer; and TC, tumor cell

**Fig. 2:**
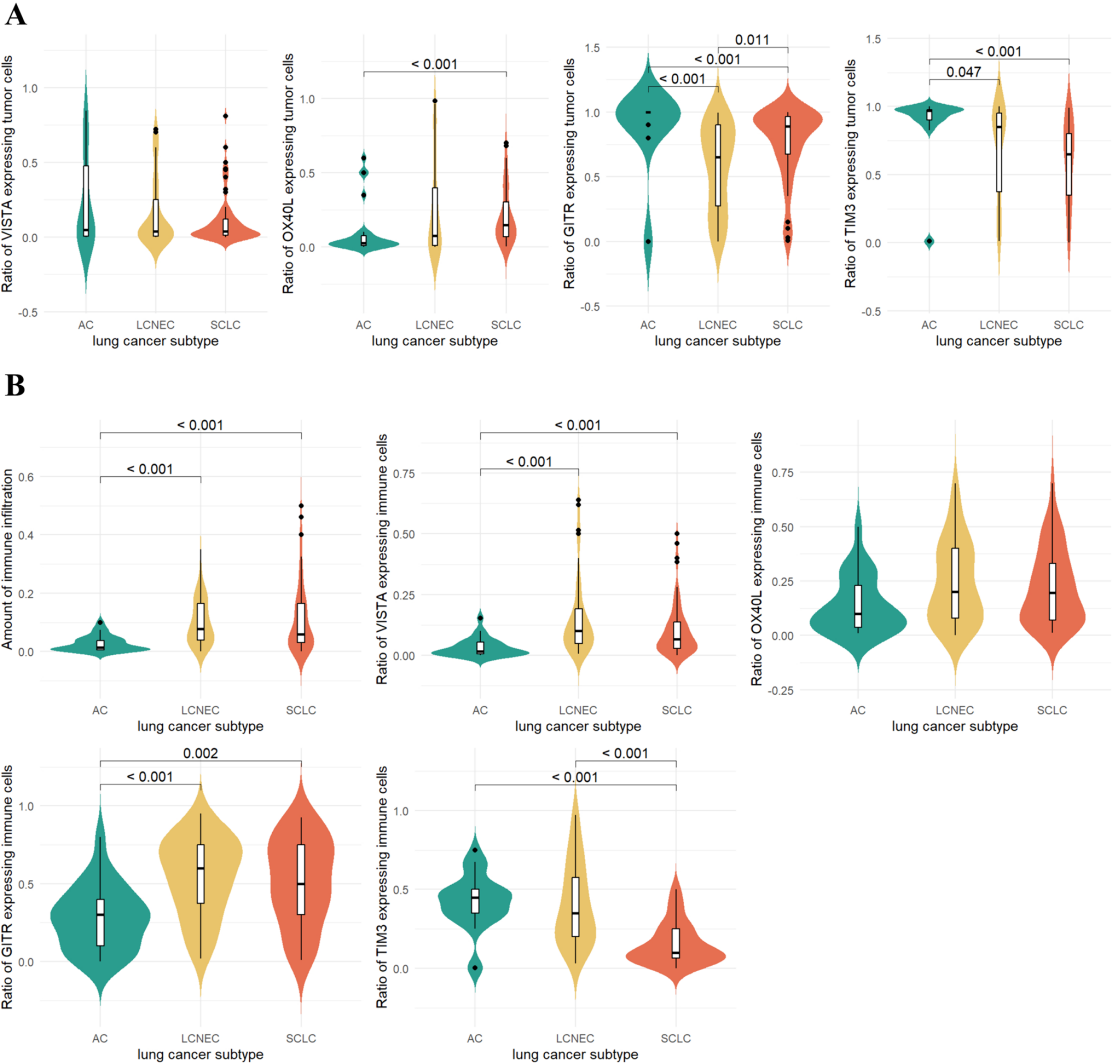
Expression levels of potential immunotherapy targets by TCs (**A**) and ICs (**B**) in different LNEN subtypes. The color-filled curves show the estimated normalized probability density function of the data. Overlaid box plots demonstrate the same distributions, and box edges represent the first (Q1) and third (Q3) quartiles, with the inner line showing the median value. Whiskers extend to 1.5-times the interquartile range (IQR = Q3-Q1). Samples outside this range (outliers) are marked by black dots. Only significant *p* values are shown. Colors indicate the three LNEN subtypes, Green: AC, atypical carcinoid; yellow: LCNEC, large cell neuroendocrine lung cancer; and orange: SCLC, small cell lung cancer

**Fig. 3:**
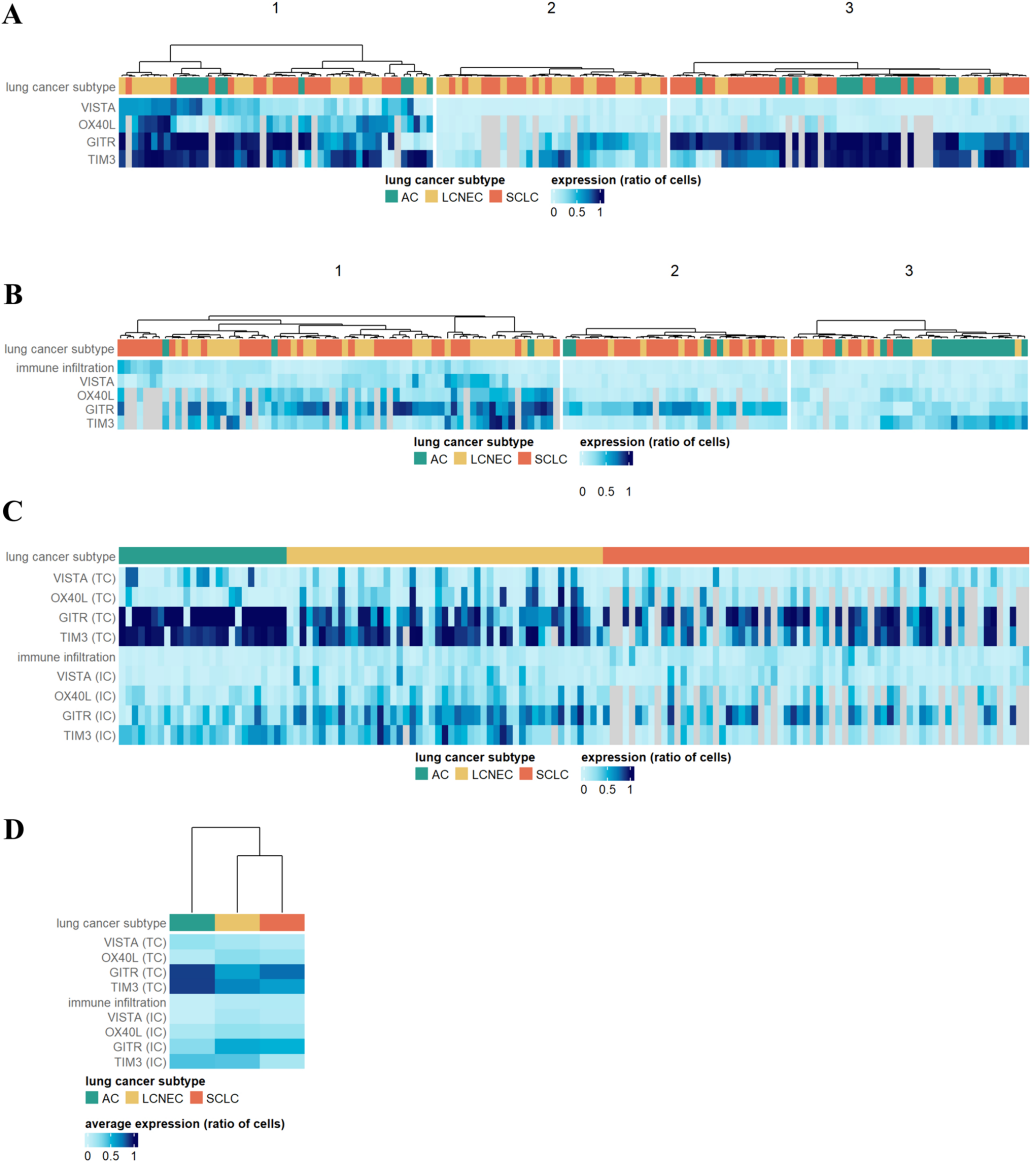
Hierarchical clustering of LNEN subtypes based on TC (**A**) and IC (**B**) VISTA, OX40L, GITR, and TIM3 expression. The color bar scale indicates the expression levels of the selected markers. LCNEC, large cell neuroendocrine lung cancer; SCLC, small cell lung cancer; AC, atypical carcinoid. **C** Heatmap of marker expression levels as defined by both TCs and ICs. **D** Heatmap of the average TC and IC marker expression levels for each subtype

Representative IHC images of each investigated marker and their TC expression levels according to LNEN subtypes are shown in Figs. 1 and 2A, respectively. Interestingly, OX40L expression of AC TCs was significantly lower than in SCLC tumors (*p* < 0.001). Meanwhile, ACs tended to demonstrate significantly higher TC GITR expression levels than SCLC or LCNEC tumors (*p* < 0.001). Of note, TC GITR expression was also considerably higher in SCLC than in LCNEC (*p* = 0.011). As for TIM3, its TC expression was significantly higher in ACs (vs. LCNEC and SCLC tumors; *p* = 0.047 and *p* < 0.001, respectively). No significant differences were observed for VISTA expression. In order to examine whether LNEN subtypes can be distinguished solely by their TC VISTA, GITR, OX40L, or TIM3 expression, we performed unsupervised hierarchical clustering. As shown in Fig. 3A, although cluster analysis differentiated three distinct subgroups with divergent immunologic phenotypes, these clusters did not conclude with the histological subtypes. When examining the clinicopathological relevance of TC marker expression, we found that grade two tumors tended to have higher GITR (*p* = 0.028) and TIM3 (*p* = 0.03) expression than grade 3 lesions (Supplementary Fig. 3A). GITR expression by TCs was also significantly higher in never-smokers than in current smokers (*p* = 0.046); yet, this is likely to be attributed to the distinct smoking habits of each LNEN patient.

### Distribution pattern of immunologic markers by ICs

To gain insights into the overall immune landscape of LNEN subtypes, we first assessed each tumor’s CD3 expression. We found that IC abundance was similar in LCNEC and SCLC samples but significantly lower in AC tumors (*p* < 0.001). ACs also expressed significantly lower levels of IC VISTA (*p* < 0.001) and GITR (*p* = 0.002) than LCNEC or SCLC tumors (Fig. 2B). Meanwhile, TIM3 expression by ICs was significantly lower in SCLCs compared to ACs (*p* < 0.001) or LCNECs (*p* < 0.001). Cluster analysis was not able to distinguish LNEN subtypes based solely on VISTA, GITR, OX40L, or TIM3 expression by ICs (Fig. 3B). With regard to clinicopathological features (Supplementary Fig. 3B), centrally located tumors tended to have significantly lower levels of immune infiltration (*p* < 0.001), and their ICs expressed significantly lower levels of VISTA and TIM3 than peripheral tumors (*p* < 0.001). Moreover, we also found that necrotic tumors presented with a significantly greater amount of immune infiltration than non-necrotic lesions (*p* = 0.027). Tumors with high peritumoral inflammation displayed increased immune infiltration compared to tumors with medium or low levels of peritumoral inflammation.

### Correlation between the expression patterns of immune-related markers defined by TCs and ICs

As shown in Supplementary Fig. 4, TC OX40L and TIM3 expression correlated with VISTA expression (*R* = 0.4928, *p* < 0.0001 and R = 0.3083, *p* = 0.0245, respectively). A positive linear correlation was also found between TC TIM3 and GITR expressions (*R* = 0.4658, *p* < 0.0001). We found that the IC GITR expression correlated with both TC GITR expression and IC OX40L expression (*R* = 0.5416, *p* = 0.0233 and *R* = 0.5678, *p* = 0.011, respectively).

To assess the impact of VISTA, OX40L, GITR, and TIM3 expressions on CD3 distribution, we correlated IC CD3 expression with both IC and TC expressions of the markers above. As shown in Supplementary Fig. 5, none of the examined immunotherapy targets correlated significantly with CD3 expression; yet a tendency toward positive linear correlation could be observed in the case of IC GITR and VISTA expressions.

### LNEN subtype-specific immunologic landscape revealed by principal component analysis

**Fig. 4:**
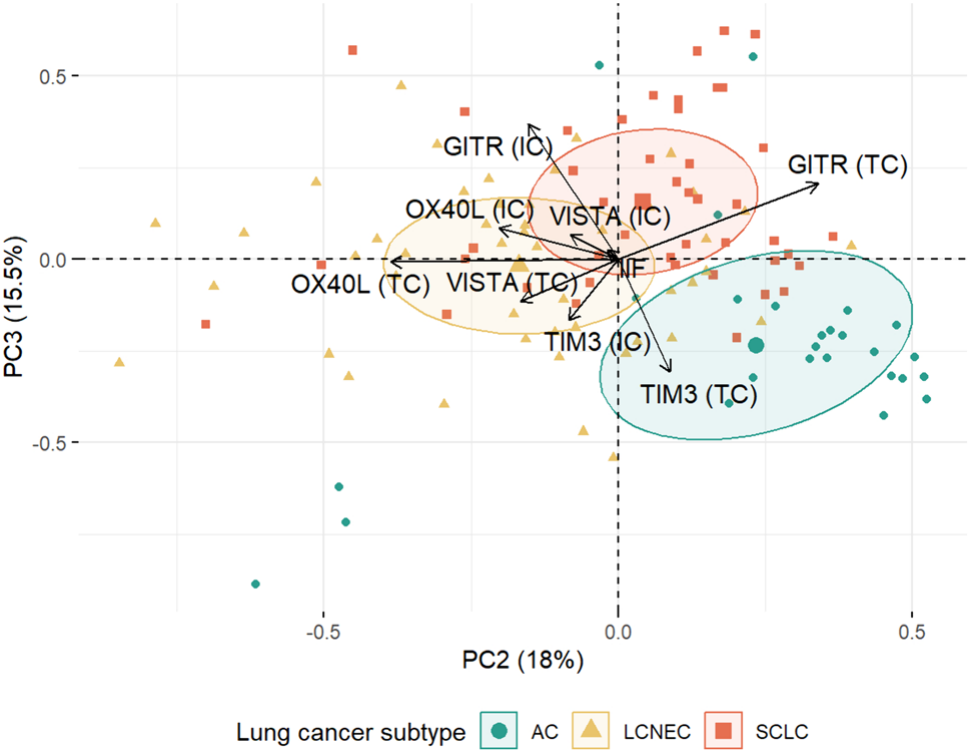
Principal component analysis of LNEN marker expression. ACs can be distinguished from LCNEC and SCLC tumors based on their IC and TC marker expression. ACs express high TC TIM3 and GITR levels and low IC GITR levels. TCs and ICs of SCLC lesions express high levels of GITR, and their ICs express low levels of TIM3. Percentage values on axis labels indicate the percentage of explained variance by the given principal component. ICs of LCNEC tumors express high levels of GITR and TIM3. AC, atypical carcinoid; LCNEC, large cell neuroendocrine lung cancer; SCLC, small cell lung cancer; and PC, principal component

Principal component analysis showed that 72% of the variance in the data is explained by the first three PCs. Upon further investigation, PC1 did not seem to effectively separate patients based on LNEN subtype, and thus, we projected all data points and original variables into the space spanned by PC2 and PC3 (Fig. 4). The figure displays that by using PCA, ACs can be distinguished from both LCNEC and SCLC tumors based on their IC and TC marker expression. In this context, (1) ACs express high levels of TC TIM3 and GITR, and low levels of IC GITR; (2) both TCs and ICs of SCLCs express high levels of GITR, and their ICs express low levels of TIM3; and (3) ICs of LCNECs express high levels of GITR and TIM3. The trends for expression levels of each sample, grouped by their histologic subtype, are shown in Fig. 3C. Figure 3D highlights the average expression pattern for each type, underlining previous observations of the most typical features.

### Overall immunological phenotype distinguishes LNEN tumors

**Fig. 5:**
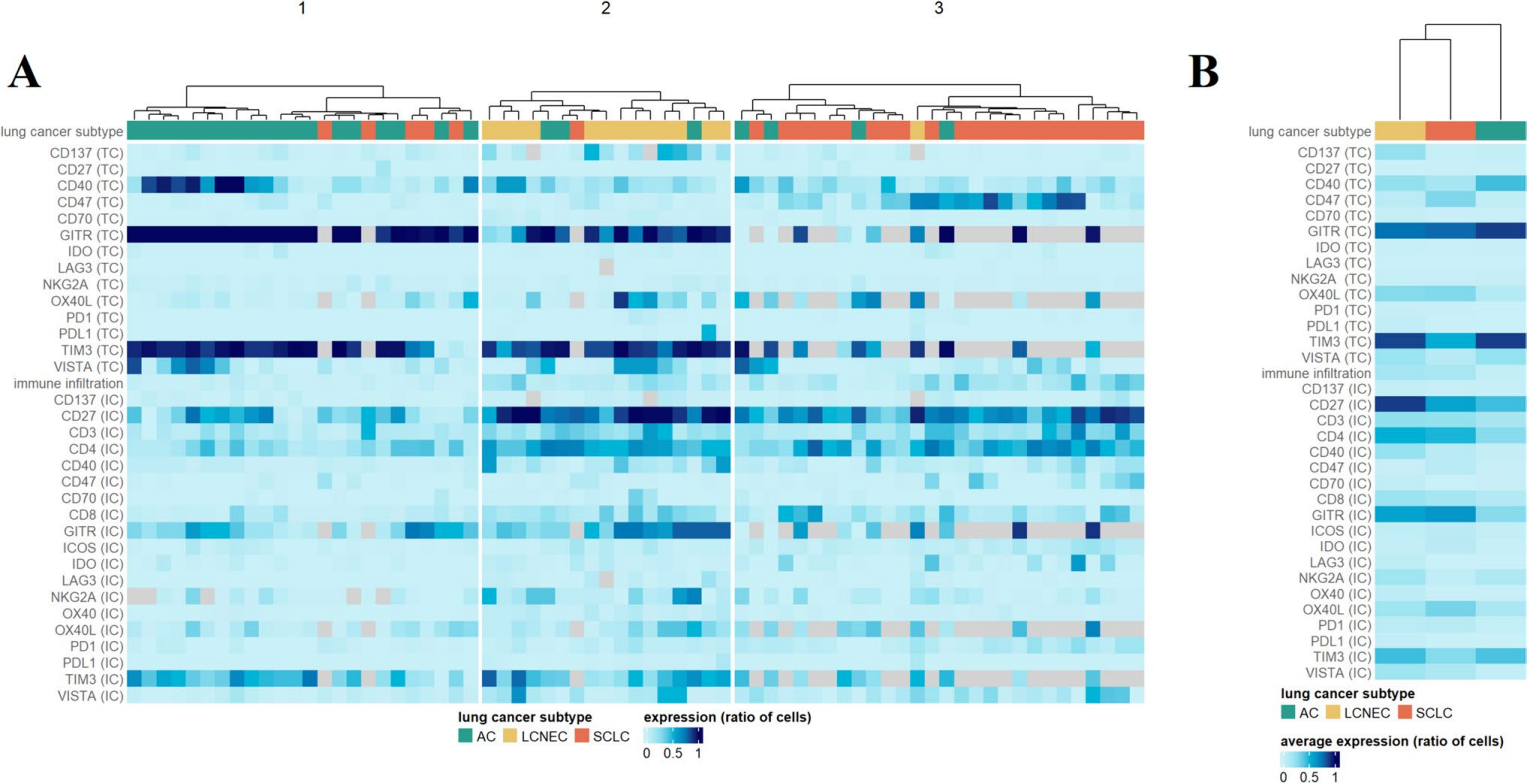
**A** Hierarchical clustering of LNENs based on the TC expression of immune-related markers. The color bar scale indicates the expression levels of CD137, CD27, CD3, CD4, CD40, CD47, CD70, CD8, GITR, ICOS, IDO, LAG3, NKG2A, OX40, OX40L, PD-1, PD-L1, TIM3, VISTA. LCNEC, large cell neuroendocrine carcinoma; SCLC, small cell lung cancer; AC, atypical carcinoid **B** Heatmap of the average TC expression levels of the selected immune-related markers

In our previous study [24], we investigated the expression pattern of 15 immune-related markers (PD-1, CD27, CD4, CD47, ICOS, LAG3, OX40, PD-L1, IDO, CD70, CD137, CD3, CD40, NKG2A, CD8) using a representative number of AC, LCNEC, and SCLC samples. Since 69 cases of the prior patient cohort overlapped with the cohort for this study, the datasets were merged in order to examine whether the overall marker expression distinguishes LNEN subtypes. Unsupervised clustering revealed unique marker expression patterns in the different histological samples. Importantly, the results demonstrate that the applied immune-related markers are highly effective in classifying tumors into their respective subgroups (Fig. 5A). Figure 5B demonstrates the average expression patterns of examined markers with regard to LNEN subtypes.

### Association between immune marker expression levels and survival

Patients were divided into low- and high-expressing categories based on the median expression levels of the examined four immune-related markers. Although TC marker expressions did not influence the OS significantly, patients with high TC TIM3 expression tended to have improved survival outcomes compared to those with low TC TIM3 levels (*p* = 0.08) (Supplementary Fig. 6A). Infiltrating ICs did not have a significant prognostic implication either (Supplementary Fig. 6B). As for IC marker expression, we found that high TIM3 expression was associated with significantly improved survival outcomes (vs. low TIM3 expression, *p* = 0.021). Meanwhile, low IC GITR expression corresponded to an increased OS with a borderline significant trend (*p* = 0.064). Lastly, we performed multivariate Cox-regression analysis to decipher the clinical parameters influencing OS (Supplementary Fig. 7). Of all examined parameters, diabetes (*p* = 0.003), histological type (AC vs. LCNEC, *p* = 0.061 and AC vs. SCLC, *p* = 0.016), and tumor grade (*p* = 0.045) influenced the OS independently. Of note, the independent prognostic relevance of IC TIM3 and GITR expression remained borderline significant (*p* = 0.057 and *p* = 0.071, respectively).

## Discussion

The last twenty years of lung cancer research have clarified that the immune infiltration of the tumorous and peritumoral regions significantly influences the fate of malignant lesions [41, 42]. Hence, gaining a better understanding of the TIM and the interactions between tumors and ICs is essential for improving the efficacy of targeted therapies and immunotherapy. As for the latter, the recent advances in immunotherapy in other tumor entities have not yet provided an equal breakthrough in LNENs [43]. A comprehensive understanding of the TIM is, therefore, crucial for efficacious treatment protocols in the future [44, 45]. Here, we investigated the expression pattern and clinicopathological relevance of four novel immunotherapy targets in surgically resected LNENs.

Currently, only a few predictive and prognostic markers have been identified in neuroendocrine neoplasms. Orthopedia homeobox protein (OTP) is a promising marker for pulmonary carcinoids. Indeed, OTP and the adhesion molecule CD44 have been described as potential prognostic markers; nevertheless, their exact impact on OS and immunotherapeutic efficacy is still controversial [46–48]. In contrast, mutation in the MEN1 (multiple endocrine neoplasia 1) gene has clearly been shown to be a poor prognostic factor in pulmonary carcinoids [49]. LCNEC has recently been classified into two major groups based on genomic and transcriptomic levels. LCNEC I is characterized by mutations in retinoblastoma protein 1 (*Rb1*) and tumor protein 53 (*TP53*), similar to SCLC. In contrast, LCNEC II exhibits alterations in NSCLC-type serine/threonine kinase 11 (*STK11*), Kelch-like ECH-associated protein 1 (*KEAP1*), and Kirsten rat sarcoma virus (*KRAS*); these are commonly seen in lung adenocarcinomas too. These molecular findings support the theory that LCNEC is a “mixed basket” of tumors with different origins [50–52]. SCLC is a particularly aggressive entity and was long considered a homogenous tumor. Recent data, however, demonstrated that SCLC tumors could be sub-segmented into distinct molecular subtypes based on the expression pattern of specific transcription factors (achaete-scute homologue 1 (ASCL1), neurogenic differentiation factor 1 (NEUROD1), POU Class 2 homeobox 3 (POU2F3)) and inflammatory characteristics. These subtypes are characterized by varying morphology, therapeutic responsiveness, and prognosis [12, 53–55].

VISTA is a membrane protein, expressed usually by myeloid cells, granulocytes, and T cells, and it functions as a negative checkpoint ligand for antigen-presenting cells and T cells [56]. Previous studies concerning other malignancies (e.g., lung, kidney, colorectal, endometrial and ovarian cancers) have described VISTA to be expressed by lymphocytes in the tumor microenvironment as well as by the TCs [57, 58]. Its prognostic significance is rather controversial, since high VISTA expression is associated with improved OS in epithelioid mesothelioma, but with worse survival outcomes in colorectal tumors [59–61]. In the present study, VISTA expression did not have a significant impact on OS. However, ICs in LCNECs and SCLCs expressed VISTA to a greater extent than in AC tumors. We postulate that VISTA might be highly expressed in malignant lesions as it inhibits T lymphocyte function, and therefore, reduces the antitumor response. Thus, VISTA might be an effective treatment target in these highly malignant lesions. In fact, experiments in murine models have confirmed that VISTA inhibition increases the number of T lymphocytes and boosts their function in the tumor environment [56]. A phase 1 clinical trial is currently investigating the efficacy of an anti-VISTA monoclonal antibody (JNJ-61610588; NCT02671955) in various solid tumors. Meanwhile, another ongoing multicenter study examines the long-term effects of CA-170, a PD-L1/PD-L2 and VISTA inhibition in solid tumors and lymphomas [56, 62–65].

The OX40 ligand “OX40L” is an immune checkpoint modulator primarily expressed on activated APCs, dendritic cells, B cells, and macrophages. [66] The attachment of OX40 and OX40L enhances the survival of CD4^+^ and CD8^+^ cells which increases tumor-specific responses of effector T cells in tumors and neutralizes the suppressive effects of *T*_reg_ cells. [67] A recent study on NSCLC found that elevated OX40L expression is associated with higher CD4^+^ infiltration and increased OS. [68] Studies in SCLC, melanoma, and pancreatic ductal adenocarcinoma have yielded similar results. [69, 70] Our study did not show any significant survival benefit concerning OX40L expression, which might partly be explained by the low number of included cases. However, we found that AC TCs typically expressed OX40L at lower levels than other LNEN TCs. Interestingly, in our previous study [24] also conducted on LNENs, we did not find significant differences in OX40 expression concerning histological subtypes [24]. This might be suggestive of independent OX40 and OX40L expression patterns. Importantly, recent in vivo studies suggest that agonistic and antagonistic therapy toward OX40-OX40L interaction might be a potential therapeutic option [28, 66, 67].

Another attractive target for immunotherapy is GITR, a costimulatory cell surface receptor [71]. It is expressed mainly on T cells and NK cells and plays a major role in the activation of effector T cells, thereby making it an appealing target for antitumoral therapy. Indeed, triggering GITR enhances the immune system’s antitumoral response in murine models by stimulating T lymphocyte activity and inhibiting Treg cells. The first clinical trial in which the GITR agonist TRX518 was applied in solid tumors commenced in 2018. Unfortunately, the survival benefits were modest, and the primary endpoints were not met despite combining TRX518 with PD-1 and PD-L1 inhibitors [30, 31, 72, 73]. Nevertheless, several additional studies investigating GITR targeting are still ongoing [74, 75]. Increased GITR expression has been shown to be a positive prognostic factor in endometrial carcinoma and head and neck tumors, whereas the opposite has been confirmed in renal carcinoma [72, 73, 75]. In our cohort, GITR was expressed to a greater degree by AC TCs than LCNEC and SCLC TCs, and ICs in ACs expressed significantly less GITR compared to LCNEC and SCLC samples. Of note, the effects of GITR expression on tumor-infiltrating ICs also vary depending on the elapsed time. Specifically, while GITR activation initially inhibits the Treg cells and thus contributes to increased immune infiltration, activating GITR excerpts an opposite effect as time passes and inhibits the antitumor immune response [31, 34, 35, 76, 77]. Indeed, we also found in our previous study that AC tumors (where GITR expression is expected to be the highest) had considerably lower levels of tumor-infiltrating CD8^+^ and CD3^+^ lymphocytes than LCNEC or SCLC tumors with high GITR expression [24]. In terms of survival, low GITR expression in the tumor environment showed a borderline significant trend for improved survival; this trend remained similar in the Cox-regression model.

TIM3, a negative T cell regulator expressed mainly on NK cells and macrophages, was the last protein examined within the framework of the current study. Free TIM3 promotes T cell activation, but once activated, it inhibits the antitumor immune response, induces immunosuppression, and contributes to poor prognosis [32, 33, 36]. Therefore, TIM3 might emerge in the future as a promising target for inhibition [75]. Indeed, blockage of both TIM3 and PD-1 has been demonstrated to result tumor regression in preclinical models, and several clinical trials are currently exploring TIM3 inhibition in solid tumors [32, 33, 78, 79]. Interestingly, both TCs and ICs expressed TIM3 to a greater degree in ACs than in LCNEC and SCLC tumors. As for its clinical relevance, high TIM3 expression by TCs and ICs tended to be associated with improved survival. Nevertheless, patients with AC tumors generally had better prognosis than those with other LNENs, and TIM3 expression could not be validated as an independent prognosticator in our multivariate model. Still, its high expression in AC tumors makes TIM3 a potential subtype-specific immunotherapeutic target for AC patients.

Despite the evident differences in their expression pattern, cluster analysis of VISTA, OX40L, GITR, and TIM3 expression did not differentiate LNEN subtypes. Of note, however, when supplementing the current results with our findings from an overlapping cohort [24], it became apparent that LNEN tumors have widely different immune phenotypes. One particularity of this finding is that AC tumors are the least immunogenic entities among all investigated LNENs, albeit they have the highest TIM3 and GITR TC expression. These immune profiles can aid in diagnosing each histologic subset and predicting potential therapeutic responses to immune checkpoint blockade. Nevertheless, as highlighted before, VISTA, OX40L, GITR, and TIM3 expression regulate the tumor immune microenvironment through a series of complex and time-dependent processes [27, 28, 30–33, 35–37, 62, 76, 79]. These aspects should be considered when assessing their impact on intratumoral immune cell distribution and antitumor response.

Our study has some limitations that must be addressed in future prospective settings. A small fraction (4,9%) of surgically resected tissue samples were older than 15 years. While most antigens in FFPE blocks are well preserved over time [80, 81], decreasing nuclear immunosignal intensity might occur in some of these older blocks. Of note, however, during our quality check, we obtained positive staining with routine diagnostic antibodies (CD56 [38] and Ki-67[39]) even in the three oldest blocks, and we found no statically significant differences between the older and newer FFPE blocks concerning immunotherapy target expression. Another limitation was that clinical and follow-up data were not available in all cases due to the study’s retrospective nature. This hindered subsequent analyses and precluded in-depth measurements (i.e., cancer-specific survival). Albeit this lack of data did not influence our findings concerning the expression pattern of investigated markers, all results deriving from clinical data analysis warrant further validation. Furthermore, although a relatively large number of rare tumors was collected, the cohort size was still small to reach statistical significance in some instances. Lastly, the current study is rather descriptive and hypothesis-generating than evidence-based since the direct effects of immunotherapeutics could not be assessed. Accordingly, the key immunologic drivers of marker expression could not be assessed within the framework of the current study and need to be verified in future experimental settings. Of note, these limitations were partly counterbalanced because all analyses were conducted on surgically resected specimens, thus avoiding the distorting effects of intra-tumoral heterogeneity and obtaining a complete overview of the tumors’ immunologic landscape.

By investigating the expression pattern of potential immunotherapy targets in intermediate- and high-grade LNENs, the current multicenter study aimed to aid the future implementation of novel immunotherapeutic approaches. We report that high TC TIM3 expression is characteristic of AC tumors, whereas elevated TC GITR levels could be found in both ACs and SCLCs. OX40L expression by TCs is the highest in SCLCs and the lowest in ACs. IC infiltration is the least pronounced in AC lesions, and IC VISTA and GITR expressions are also considerably lower in these intermediate-grade malignancies. Altogether, these results might aid in designing clinical trials evaluating the efficacy of particular immunotherapeutics directed against VISTA, OX40L, GITR, and TIM3 in these hard-to-treat malignancies.

### Supplementary Information

Below is the link to the electronic supplementary material.

Supplementary file1 (JPG 4973 KB)

Supplementary file2 (JPG 5099 KB)

Supplementary file3 (JPG 1458 KB)

Supplementary file4 (JPG 2360 KB)

Supplementary file5 (JPG 560 KB)

Supplementary file6 (JPG 2110 KB)

Supplementary file7 (JPG 546 KB)

Supplementary file8 (DOCX 13 KB)

Supplementary file9 (DOCX 12 KB)

## Data Availability

Data were generated by the authors and are available upon reasonable request.
