# Supplementary figures and images for: Expression patterns of novel immunotherapy targets in intermediate- and high-grade lung neuroendocrine neoplasms
Source: Cancer Immunol Immunother. 2024 May 2;73(6):114. doi: 10.1007/s00262-024-03704-7 (PMC11063022; doi:10.1007/s00262-024-03704-7)

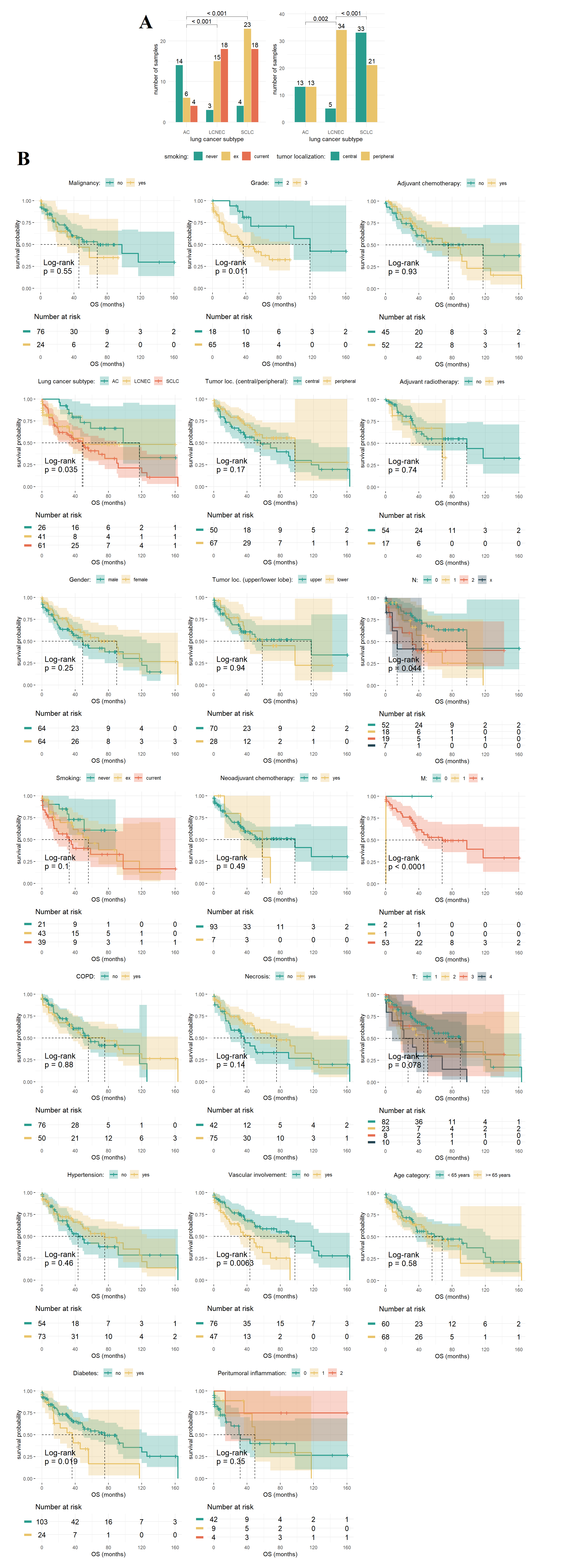

Supplement: Supplementary file 1 — Supplementary file1 (JPG 4973 KB) [file 262_2024_3704_MOESM1_ESM.jpg]

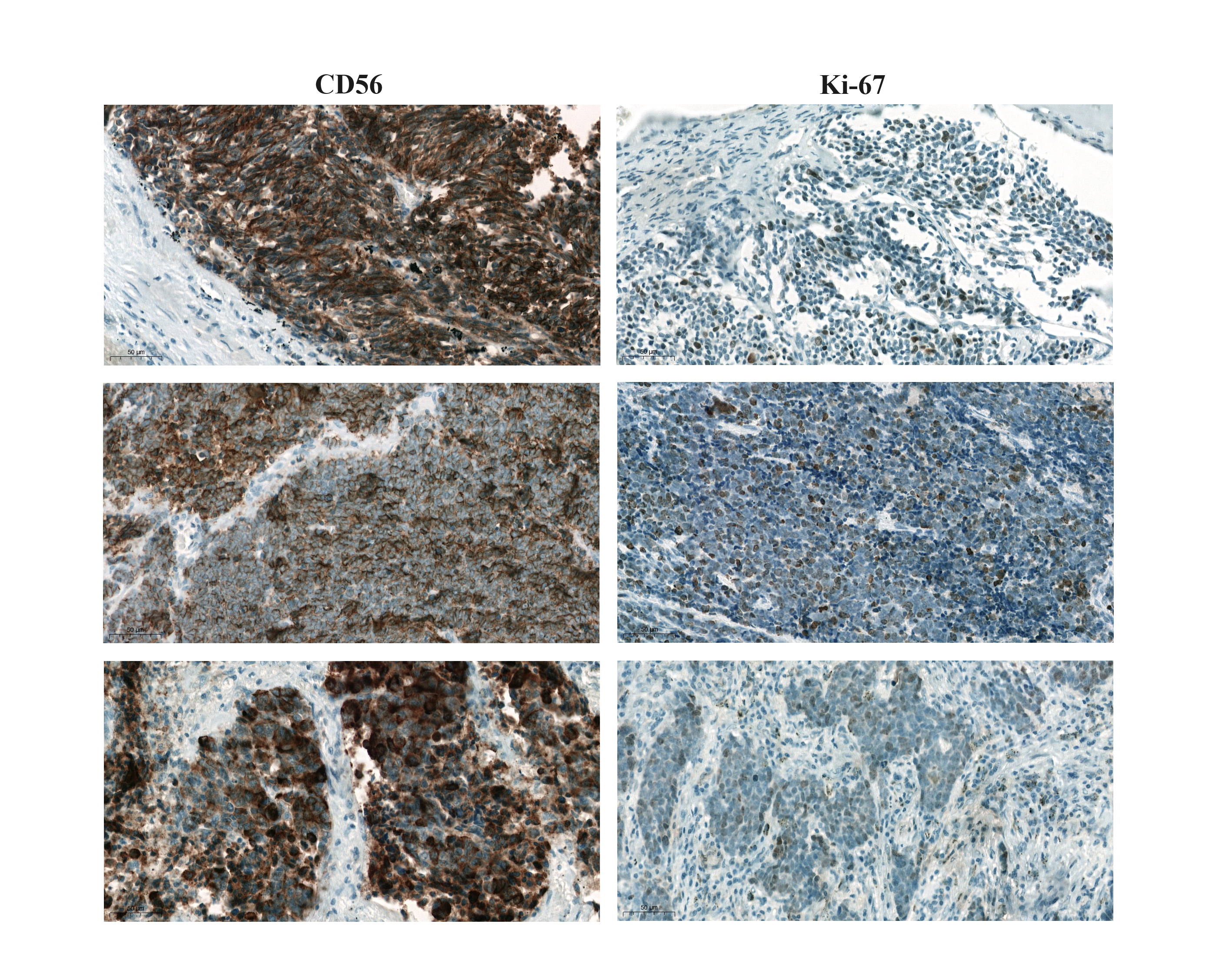

Supplement: Supplementary file 2 — Supplementary file2 (JPG 5099 KB) [file 262_2024_3704_MOESM2_ESM.jpg]

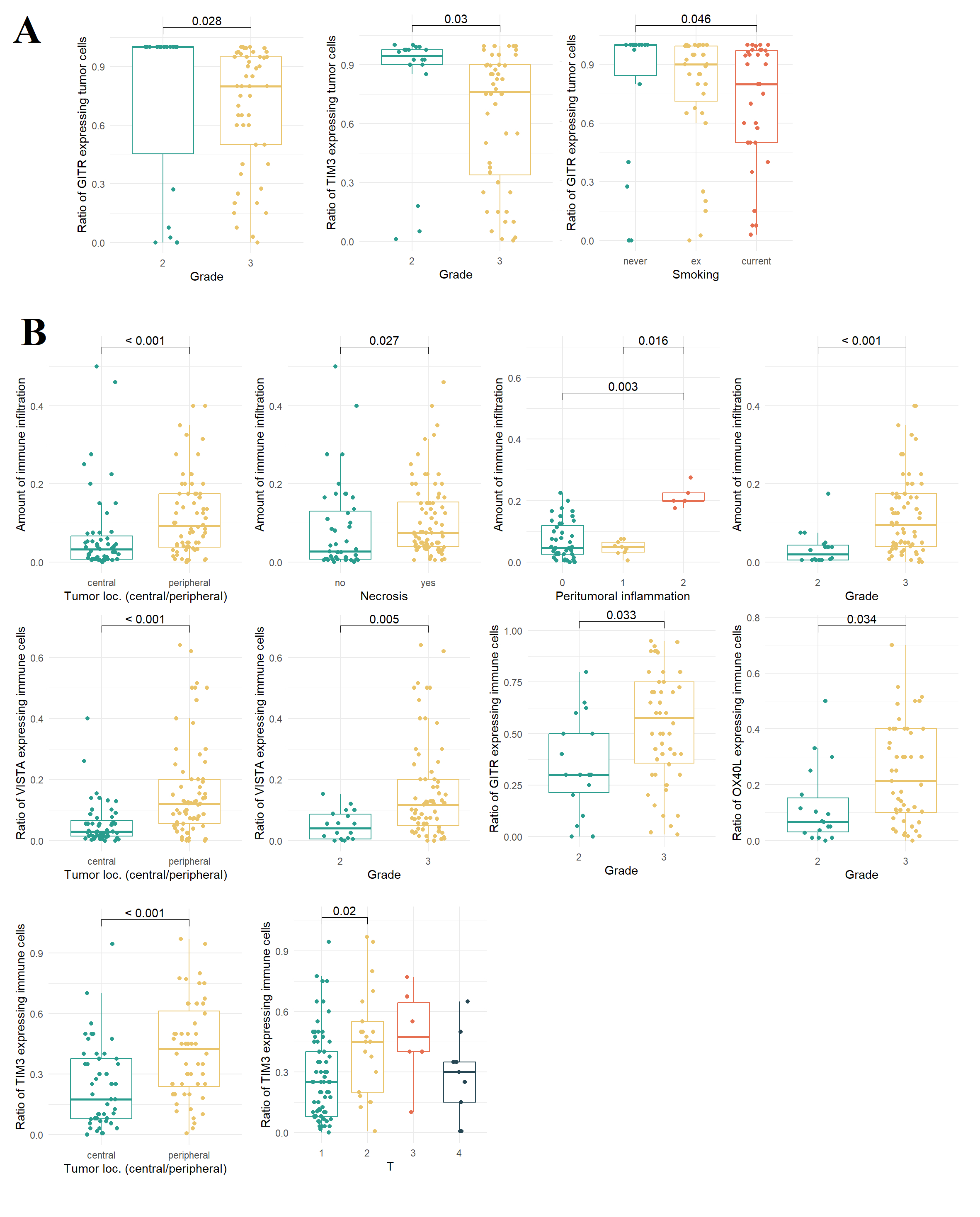

Supplement: Supplementary file 3 — Supplementary file3 (JPG 1458 KB) [file 262_2024_3704_MOESM3_ESM.jpg]

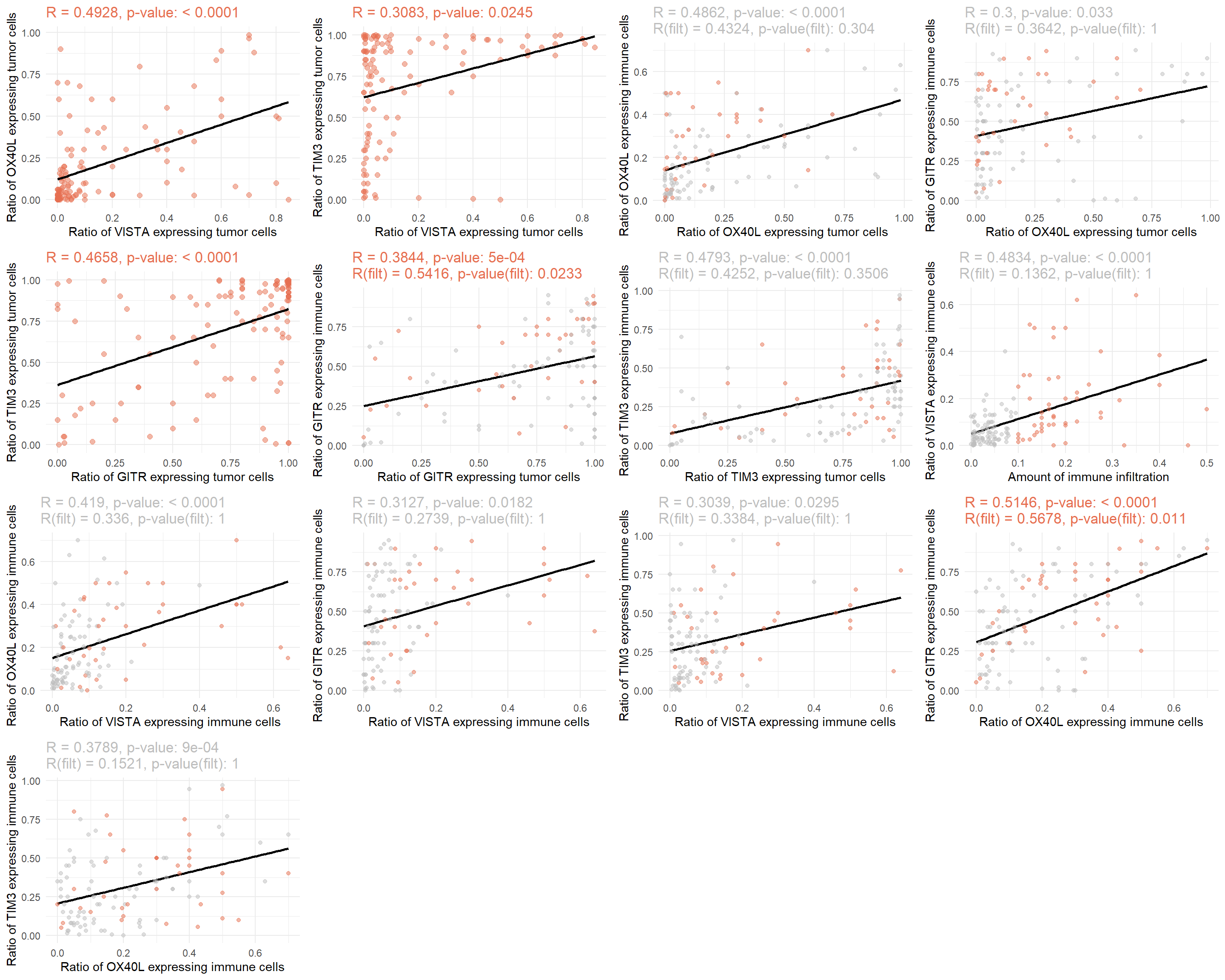

Supplement: Supplementary file 4 — Supplementary file4 (JPG 2360 KB) [file 262_2024_3704_MOESM4_ESM.jpg]

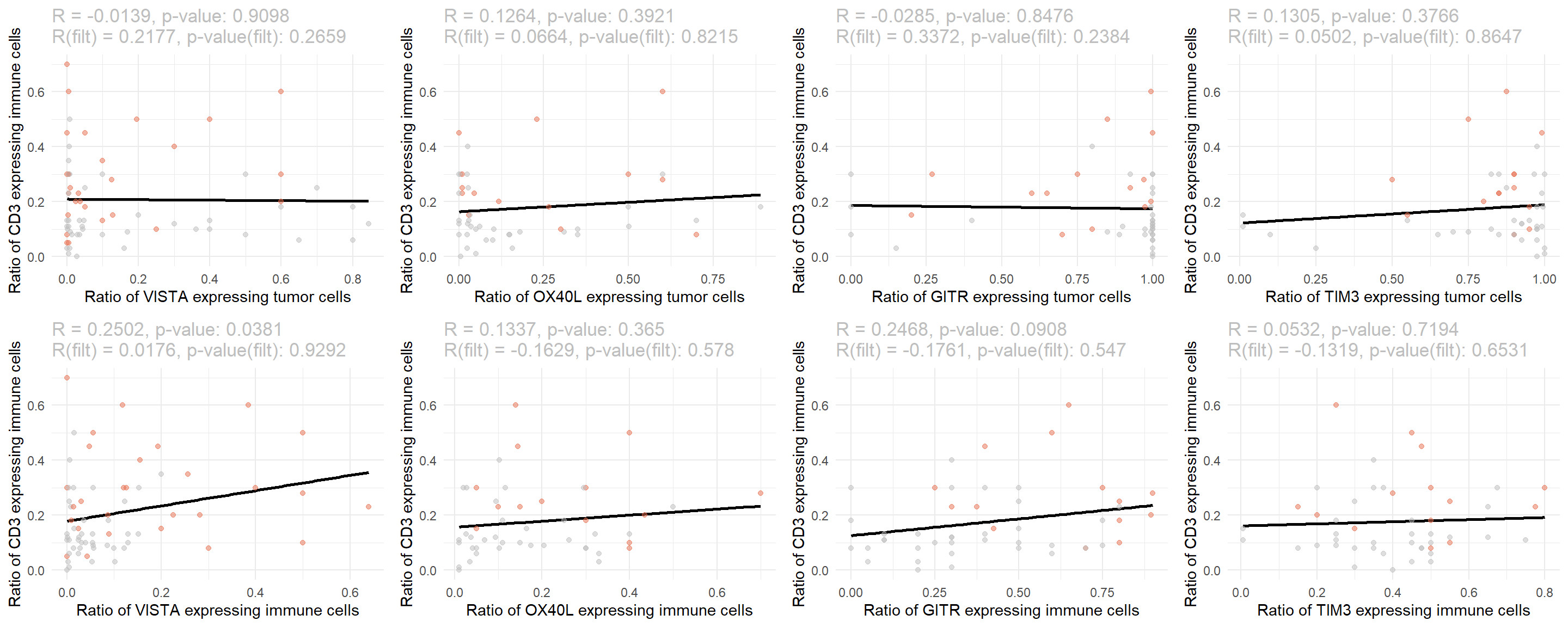

Supplement: Supplementary file 5 — Supplementary file5 (JPG 560 KB) [file 262_2024_3704_MOESM5_ESM.jpg]

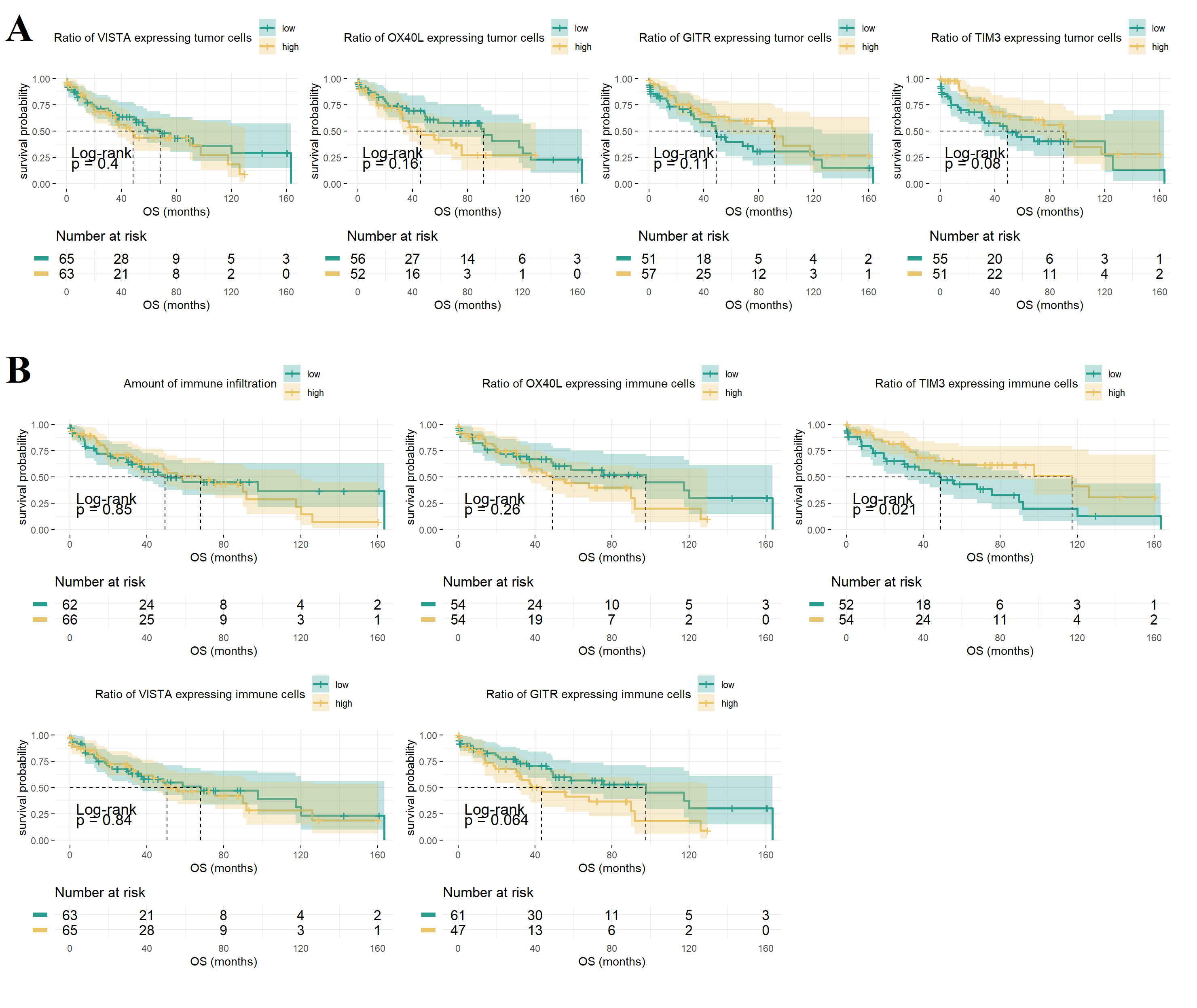

Supplement: Supplementary file 6 — Supplementary file6 (JPG 2110 KB) [file 262_2024_3704_MOESM6_ESM.jpg]

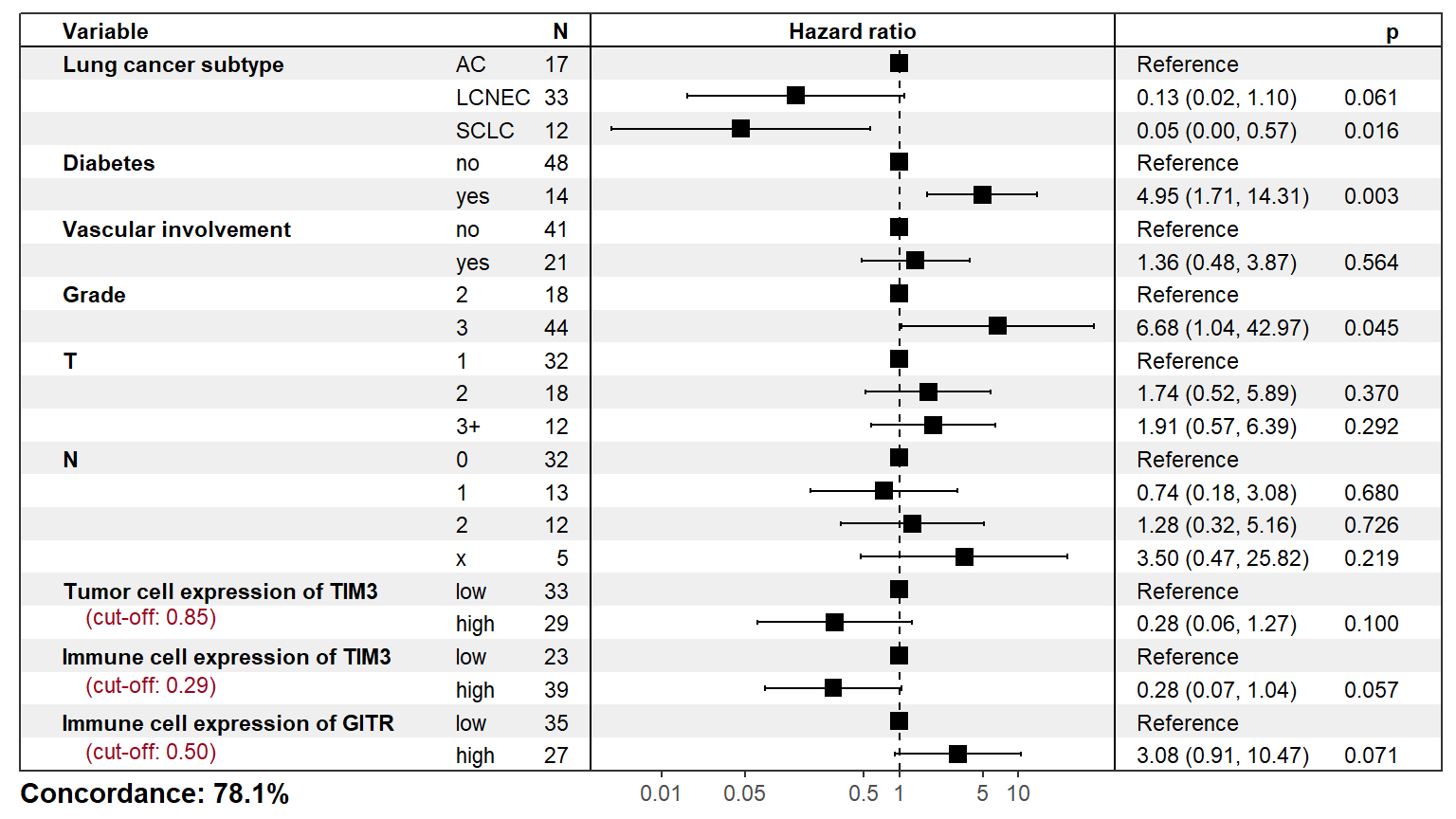

Supplement: Supplementary file 7 — Supplementary file7 (JPG 546 KB) [file 262_2024_3704_MOESM7_ESM.jpg]
