# Supplementary material for: Expression patterns of novel immunotherapy targets in intermediate- and high-grade lung neuroendocrine neoplasms
Source: Cancer Immunol Immunother. 2024 May 2;73(6):114. doi: 10.1007/s00262-024-03704-7 (PMC11063022; doi:10.1007/s00262-024-03704-7)
Supplement: Supplementary file 8 — Supplementary file8 (DOCX 13 KB) [file 262_2024_3704_MOESM8_ESM.docx]

| **Antibody** | **Company** | **Catalog nr.** | **Host** | **Dilution** | **Antigen retrieval** |
| --- | --- | --- | --- | --- | --- |
| **TIM3** | Abcam, Boston, USA | ab185703 | Rabbit | 1:100 | Citrate (pH=6.0) |
| **VISTA** | Sino Biological, Beijing, China | 13482-T24 | Rabbit | 1:500 | Citrate (pH=6.0) |
| **GITR** | Thermo Fisher Scientific, Waltham, MA, USA | PA5-46810 | Rabbit | 1:100 | Citrate (pH=6.0) |
| **OX40L** | Thermo Fisher Scientific, Waltham, MA, USA | 11-1347-42/ACT35 | Rabbit | 1:100 | Citrate (pH=6.0) |
| **CD3** | Leica, Wetzlar, Germany | PA0553/LN10 | Mouse | Ready-to-use | Tris-EDTA (pH=9.0) |

**Supplementary table S1.** Antibodies used for immunohistochemistry.
