# Supplementary material for: Expression patterns of novel immunotherapy targets in intermediate- and high-grade lung neuroendocrine neoplasms
Source: Cancer Immunol Immunother. 2024 May 2;73(6):114. doi: 10.1007/s00262-024-03704-7 (PMC11063022; doi:10.1007/s00262-024-03704-7)
Supplement: Supplementary file 9 — Supplementary file9 (DOCX 12 KB) [file 262_2024_3704_MOESM9_ESM.docx]

**Supplementary Figure 1.** (A) Statistically significant associations between LNEN subtypes and clinical parameters. The horizontal axis shows the investigated clinical variables, whereas the number of samples is displayed on the vertical axis. (B) Kaplan-Meier survival estimates according to clinicopathological parameters.

**Supplementary Figure 2.** Representative IHC images of the three oldest FFPE samples stained with CD56 and Ki-67. Representative images were captured with a 40x objective lens.

**Supplementary Figure 3.** Statistically significant associations between the TC (A) and IC (B) expression levels of potential immunotherapy targets and clinicopathological parameters. Only those associations are plotted that remained significant after multiple testing corrections, p-values refer to pairwise comparisons.

**Supplementary Figure 4**. Correlation between TC and IC VISTA, OX40L, GITR and TIM3 expression. Only associations that remained significant after Bonferroni-correction are shown. All p-values are adjusted for multiple comparisons. Results were obtained for a filtered dataset including only samples with an immune infiltration of 10% or larger, indicated with „(filt)”. If the observed correlation remained significant on the filtered dataset, the results are highlighted in orange. On panels with multiple marker colors, orange indicates samples for which immune infiltration exceeded 10%.

**Supplementary figure 5.** Correlation patterns between tumor cell and immune cell VISTA, OX40L, GITR, and TIM3 expression and immune cell CD3 expression in surgically resected LNENs.

**Supplementary Figure 6.** Kaplan-Meier estimates for OS concerning TC (A) and IC (B) VISTA, OX40L, GITR and TIM3 expression.

**Supplementary Figure 7.** Multivariate Cox-regression model for OS. The outcomes are presented as hazard ratios (HR) and their corresponding 95% confidence intervals (CI). N indicates the number of samples belonging to a given category. P-values show the significance of the associations. ICs, immune cells; OS, overall survival; TCs, tumor cells.
